# A Bibliometric Analysis of the Top 100 Cited Articles on Hepatic Magnetic Resonance Imaging

**DOI:** 10.7759/cureus.2733

**Published:** 2018-06-04

**Authors:** Waqas Ullah, Hafez Mohammad A Abdullah, Ejaz Ahmad, Mamoon Ur Rashid, Muhammad Bashir, Asad Ur Rahman, Asrar Ahmad, Abu Hurairah

**Affiliations:** 1 Internal Medicine, Abington Jefferson Health, Abington, USA; 2 Internal Medicine, University of South Dakota Sanford School of Medicine, Sioux Falls, USA; 3 Internal Medicine, Nishtar Hospital, Multan, Pakistan; 4 Internal Medicine, Florida Hospital-Orlando, Orlando, USA; 5 Internal Medicine, University of Oklahoma Health Sciences Center; 6 Gastroenterology, Cleveland Clinic Florida, Weston, USA; 7 Internal Medicine, Abington Memorial Hospital, Abington, USA; 8 Division of Gastroenterology, Department of Medicine, SUNY Downstate Medical Center, Brooklyn, NY, USA, New York, USA

**Keywords:** hepatic mri, top 100 cited articles, bibliometric analysis, bibliometrics

## Abstract

The purpose of this study is to guide the readers to the impact of the articles published on hepatic magnetic resonance imaging (MRI). We searched Scopus using 10 different search terms for hepatic MRI. The selected studies were thoroughly reviewed by two independent authors and any disagreement was sorted out by mutual consensus. The list of articles and journals was downloaded into an excel spreadsheet. Only the top 100 cited articles were selected by mutual consensus among all the authors. These articles were further read in the full-text form and were further categorized into subgroups. Three authors independently reviewed the top 100 selected articles, and subsequently data was extracted from them and analyzed. Our study showed that the highest number of top 100 cited articles on hepatic MRI were from Radiology (30 articles) followed by European Radiology (14 articles). The American Journal of Roentgenology, Radiographics, and Journal of Magnetic Resonance had seven articles each. The United States had the highest number of articles by region. Nineteen other journals contributed only one article each to the list of top 100 cited articles. The contribution of authors to the top 100 cited articles was reviewed; all the authors contributing with more than two articles to the highly cited articles are given in Table 3 in the supplementary material. The maximum number of articles were published during 2009 (14 articles), and for a five-year period, the maximum contribution was made during 2008-2013 (44 articles). Our analysis gives an insight on the frequency of citations of top articles on hepatic MRI, categorizes the subtopics, the timeline of the publications, and contributions from different geographic distributions.

## Introduction and background

Many academic institutions, private and public organizations are concerned about the quality and productivity of scholarly projects, as these are often the main parameters to prioritize resources. Bibliometric analysis is a powerful tool to determine the impact of a scholarly work on the scientific community [1]. The greater the number of the citations of an article, the greater is its contribution to the literature. Furthermore, by determining the citation frequency we can easily identify important issues and give suggestions for future research directions. However, there is a paucity of literature concerning top article citations on hepatic magnetic resonance imaging (MRI). The main purpose of this study is to guide the readers about the impact of the articles published on hepatic MRI.

## Review

In January 2017, the Scopus library database was searched for the citations of published articles on hepatic MRI using the terms “hepatic MRI, liver MRI, hepatic magnetic resonance, liver magnetic resonance, hepatic imaging, liver imaging, hepatic radiological imaging, liver radiological imaging, hepatic magnetic resonance imaging, and liver magnetic resonance imaging.” In this list, other imaging techniques such as computed tomography (CT) scan, endoscopic retrograde cholangio-pancreatography (ERCP), hepatobiliary iminodiacetic acid (HIDA) scan, and magnetic resonance cholangiopancreatography (MRCP) were not the focus of our search strategy and hence, all these articles along with other irrelevant articles were excluded. No language or time restrictions were placed on the search. Similarly, there was no restriction on the types of studies; however, studies discussing non-human subjects and case reports were excluded. The selected studies were thoroughly reviewed by two independent authors and any disagreement was sorted out by mutual consensus. The list of article and journals was downloaded into an excel spreadsheet. All articles and their corresponding journals were collected in a single search using the Scopus library. The articles were sorted on the basis of their citations using the option “Times cited” that provided us with a list of all selected articles ranked on the basis of their citation frequency. Only the top 100 cited articles were selected by mutual consensus among all the authors. These articles were further read in the full-text form and were further categorized into subgroups. Using the screening method proposed by Lim et al. [2], three authors independently reviewed the top 100 selected articles and the following data were extracted and analysed: journal name and its impact factor along with the number of citation and articles per journal, number of authors and his or her contribution along with his or her authorship position, publication year and frequency of top-cited articles in five years range, country of the first author or country where the study was originally conducted, and type of article (review or original).

A total of 100 articles were selected on the basis of their citations, the number of citations ranged from 73 to 371 with a total number of citations equal to 13558 and a median number of citations equal to 467.52 (Table 1).

**Table 1:** Top 100 cited articles on hepatic magnetic resonance imaging (MRI) MRI: magnetic resonance imaging; MR: magnetic resonance; RES: reticulo-endothelial system; CT: computed tomography; ADC: apparent diffusion coefficient; Gd-EOB-DTPA: Gadolinium Ethoxybenzyl Diethylenetriamine Pentaacetic Acid; U.S: United States; ROC: reciever operative characteristics; SPIO: superparamagnetic iron oxide; 1H-MRS: brain proton magnetic resonance spectrometry; MnDPDP: mangafodipir trisodium; OPTN: Organ Procurement and Transplantation Network; UNOS: Network for Organ Sharing; Gd-DTPA: Gadolinium Diethylenetriamine Pentaacetic Acid; 18F-FDG PET/CT: 2-deoxy-2-[fluorine-18] fluoro- D-glucose with positron emission tomography/computed tomography; OATP8: organic anion transporting polypeptide.

Rank	Article	Citations
1	Diffusion-weighted MR imaging of the liver	371
2	Focal liver lesion detection and characterization with diffusion-weighted MR imaging: Comparison with standard breath-hold T2-weighted imaging	313
3	Non-invasive assessment and quantification of liver steatosis by ultrasound, computed tomography and magnetic resonance	312
4	Ferucarbotran (Resovist): A new clinically approved RES-specific contrast agent for contrast-enhanced MRI of the liver: Properties, clinical development, and applications	294
5	Improved Detection of Focal Liver Lesions at MR Imaging: Multicenter Comparison of Gadoxetic Acid-enhanced MR Images with Intraoperative Findings	278
6	Hepatocellular carcinoma and dysplastic nodules in patients with cirrhosis: Prospective diagnosis with MR imaging and explantation correlation	262
7	Diagnostic efficacy of gadoxetic acid (Primovist)-enhanced MRI and spiral CT for a therapeutic strategy: Comparison with intraoperative and histopathologic findings in focal liver lesions	258
8	Characterization of focal liver lesions by ADC measurements using a respiratory triggered diffusion-weighted single-shot echo-planar MR imaging technique	250
9	Accurate preoperative evaluation of liver mass lesions without fine- needle biopsy	243
10	State of the art: Contrast agents for MR imaging of the liver	239
11	Perfusion imaging of the liver: Current challenges and future goals	220
12	MR imaging of hepatocellular carcinoma in the cirrhotic liver: Challenges and controversies	219
13	Accurate differentiation of focal nodular hyperplasia from hepatic adenoma at gadobenate dimeglumine-enhanced MR imaging: Prospective study	219
14	Magnetic resonance imaging of hepatic fibrosis: Emerging clinical applications	209
15	Fatty infiltration of the liver: Quantification by 1H localized magnetic resonance spectroscopy and comparison with computed tomography	200
16	Quantitative assessment of liver fat with magnetic resonance imaging and spectroscopy	192
17	Blood flow and liver imaging	189
18	Manganese ferrite nanoparticle micellar nanocomposites as MRI contrast agent for liver imaging	182
19	Hepatobiliary-specific MR contrast agents: Role in imaging the liver and biliary tree	178
20	Hepatobiliary contrast agents for contrast-enhanced MRI of the liver: Properties, clinical development and applications	177
21	Focal lesions in cirrhotic explant livers: Pathological evaluation and accuracy of pretransplantation imaging examinations	177
22	Safety, tolerance, biodistribution, and MR imaging enhancement of the liver with gadobenate dimeglumine: Results of clinical pharmacologic and pilot imaging studies in nonpatient and patient volunteers	177
23	Effects of nanoparticle size on cellular uptake and liver MRI with polyvinylpyrrolidone-coated iron oxide nanoparticles	174
24	MR imaging in patients with suspected liver metastases: Value of liver-specific contrast agent Gd-EOB-DTPA	172
25	Gadoxetate disodium-enhanced MRI of the liver: Part 1, protocol optimization and lesion appearance in the noncirrhotic liver	168
26	Hepatic arterial-phase dynamic gadolinium-enhanced MR imaging: Optimization with a test examination and a power injector	168
27	Imaging of hepatic steatosis	165
28	Efficacy and safety of MR imaging with liver-specific contrast agent: U.S. multicenter phase III study	155
29	Hepatic MRI for fat quantitation: Its relationship to fat morphology, diagnosis, and ultrasound	155
30	Hepatic lesion detection and characterization: Value of nonenhanced MR imaging, superparamagnetic iron oxide-enhanced MR imaging, and spiral CT - ROC analysis	154
31	Detection of liver metastases from endocrine tumors: A prospective comparison of somatostatin receptor scintigraphy, computed tomography, and magnetic resonance imaging	151
32	Diagnostic performance and description of morphological features of focal nodular hyperplasia in Gd-EOB-DTPA-enhanced liver magnetic resonance imaging: Results of a multicenter trial	147
33	Focal liver lesions: Evaluation of the efficacy of gadobenate dimeglumine in MR imaging - A multicenter phase III clinical study	144
34	Detection and Characterization of Focal Liver Lesions: A Japanese Phase III, Multicenter Comparison between Gadoxetic Acid Disodium-Enhanced Magnetic Resonance Imaging and Contrast-Enhanced Computed Tomography Predominantly in Patients with Hepatocellular Carcinoma and Chronic Liver Disease	143
35	Hepatic MRI with SPIO: Detection and characterization of focal liver lesions	143
36	Hepatocellular carcinoma: Hepatocyte-selective enhancement at gadoxetic acid-enhanced MR imaging - Correlation with expression of sinusoidal and canalicular transporters and bile accumulation	140
37	Hepatic MR imaging with a dynamic contrast-enhanced isotropic volumetric interpolated breath-hold examination: Feasibility, reproducibility, and technical quality	140
38	Quantification of hepatic steatosis with T1-independent, T2*-corrected MR imaging with spectral modeling of fat: Blinded comparison with MR spectroscopy	139
39	The diagnosis and management of benign hepatic tumors	139
40	Non-invasive means of measuring hepatic fat content	137
41	Diffusion-weighted MR imaging with single-shot echo-planar imaging in the upper abdomen: Preliminary clinical experience in 61 patients	137
42	Fatty liver: Imaging patterns and pitfalls	136
43	The diagnostic accuracy of US, CT, MRI and 1H-MRS for the evaluation of hepatic steatosis compared with liver biopsy: A meta-analysis	135
44	Gadoxetic acid-enhanced magnetic resonance imaging for differentiating small hepatocellular carcinomas (≤2 cm in diameter) from arterial enhancing pseudolesions: Special emphasis on hepatobiliary phase imaging	134
45	Epithelioid hemangioendothelioma of the liver: Imaging findings with pathologic correlation	126
46	Liver tumor characterization: Comparison between liver-specific gadoxetic acid disodium-enhanced MRI and biphasic CT - A multicenter trial	124
47	Role of Radiologic Modalities in the Management of Non-alcoholic Steatohepatitis	119
48	MR imaging of liver fibrosis: Current state of the art	117
49	Hepatic iron concentration: Noninvasive estimation by means of MR imaging techniques	111
50	Imaging-based quantification of hepatic fat: Methods and clinical applications	109
51	Cirrhosis or chronic hepatitis: Evaluation of small (≤2-cm) early-enhancing hepatic lesions with serial contrast-enhanced dynamic MR imaging	107
52	Magnetic resonance imaging and spectroscopy for monitoring liver steatosis	105
53	Can imaging modalities diagnose and stage hepatic fibrosis and cirrhosis accurately?	103
54	Detection of nodules in liver cirrhosis: Spiral computed tomography or magnetic resonance imaging? A prospective study of 88 nodules in 34 patients	103
55	Performance of imaging modalities in diagnosis of liver metastases from colorectal cancer: A systematic review and meta-analysis	101
56	Focal malignant hepatic lesions: MR imaging enhanced with gadolinium benzyloxypropionictetra- acetate (BOPTA) - Preliminary results of phase II clinical application	100
57	Abdominal iron deposition: Metabolism, MR findings, and clinical importance	100
58	New concepts of amebic liver abscess derived from hepatic imaging, serodiagnosis, and hepatic enzymes in 67 consecutive cases in san diego	100
59	Improved focal liver lesion detection: Comparison of single-shot diffusion-weighted echoplanar and single-shot T2-weighted turbo spin echo techniques	97
60	Imaging of hepatic cirrhosis	97
61	Assessment of hepatic steatosis in patients undergoing liver resection: Comparison of US, CT, T1-weighted dual-echo MR imaging, and point-resolved 1H MR spectroscopy	96
62	Advanced MRI methods for assessment of chronic liver disease	96
63	Respiratory-triggered versus breath-hold diffusion-weighted MRI of liver lesions: Comparison of image quality and apparent diffusion coefficient values	96
64	Clinical value of MRI liver-specific contrast agents: A tailored examination for a confident non-invasive diagnosis of focal liver lesions	96
65	Application of superparamagnetic iron oxide to imaging of hepatocellular carcinoma	95
66	Focal hepatic lesions: Diagnostic value of enhancement pattern approach with contrast-enhanced 3D gradient-echo MR imaging	93
67	Diffusion-weighted MRI provides additional value to conventional dynamic contrast-enhanced MRI for detection of hepatocellular carcinoma	92
68	Diagnosis of hepatic metastasis: Comparison of respiration-triggered diffusion-weighted echo-planar MRI and five T2-weighted turbo spin-echo sequences	92
69	Detection of colorectal liver metastases: A prospective multicenter trial comparing unenhanced MRI, MnDPDP-enhanced MRI, and spiral CT	92
70	Contrast-enhanced MR imaging of the liver	92
71	New OPTN/UNOS policy for liver transplant allocation: Standardization of liver imaging, diagnosis, classification, and reporting of hepatocellular carcinoma	91
72	Diffusion-weighted imaging of the liver: Comparison of navigator triggered and breathhold acquisitions	91
73	Hepatocellular carcinoma in patients with chronic liver disease: Comparison of SPIO-enhanced MR imaging and 16-detector row CT	91
74	Small arterial-portal venous shunts: A cause of pseudolesions at hepatic imaging	89
75	Focal liver lesions: MR imaging-pathologic correlation	86
76	MRI and ultrasound for hepatic fat quantification: Relationships to clinical and metabolic characteristics of pediatric nonalcoholic fatty liver disease	85
77	Low-dose gadobenate dimeglumine versus standard dose gadopentetate dimeglumine for contrast-enhanced magnetic resonance imaging of the liver: An intra-individual crossover comparison	85
78	Nontumorous hepatic arterial-portal venous shunts: MR imaging findings	85
79	Magnetic resonance imaging and spectroscopy accurately estimate the severity of steatosis provided the stage of fibrosis is considered	84
80	Transplantation for hepatocellular carcinoma and cirrhosis: Sensitivity of magnetic resonance imaging	84
81	Liver lesion detection, characterization, and effect on patient management: Comparison of single-phase spiral CT and current MR techniques	84
82	Gadolinium‐labeled liposomes containing various amphiphilic Gd‐DTPA derivatives: Targeted MRI contrast enhancement agents for the liver	84
83	Noninvasive Assessment of Hepatic Steatosis	83
84	MR contrast agents for liver imaging: What, when, how	83
85	Fat in the liver: Diagnosis and characterization	83
86	Quantitative evaluation of liver function with MRI using Gd-EOB-DTPA	83
87	The use of 18F-FDG PET/CT in colorectal liver metastases - Comparison with CT and liver MRI	82
88	Cirrhosis-associated hepatocellular nodules: Correlation of histopathologic and MR imaging features	82
89	CT and MR imaging of hepatic metastases	82
90	The uptake transporter OATP8 expression decreases during multistep hepatocarcinogenesis: Correlation with gadoxetic acid enhanced MR imaging	81
91	Staging hepatic fibrosis: Comparison of gadoxetate disodium-enhanced and diffusion-weighted mr imaging - Preliminary observations	81
92	T1-independent, T2*-corrected chemical shift based fat-water separation with multi-peak fat spectral modeling is an accurate and precise measure of hepatic steatosis	80
93	Black-blood diffusion-weighted EPI acquisition of the liver with parallel imaging: Comparison with a standard T2-weighted sequence for detection of focal liver lesions	78
94	Characterization of liver lesions with mangafodipir trisodium-enhanced MR imaging: Multicenter study comparing MR and dual-phase spiral CT	78
95	Non-invasive diagnosis of non-alcoholic fatty liver disease. A critical appraisal	77
96	Magnetic resonance imaging of the upper abdomen using a free-breathing T2-weighted turbo spin echo sequence with navigator triggered prospective acquisition correction	76
97	Comparison of gadolinium-EOB-DTPA-enhanced and diffusion-weighted liver MRI for detection of small hepatic metastases	75
98	Consensus report of the 2nd International Forum for Liver MRI.	74
99	Evaluation of the accuracy of gadobenate dimeglumine-enhanced MR imaging in the detection and characterization of focal liver lesions	74
100	Nonalcoholic fatty liver disease: MR imaging of liver proton density fat fraction to assess hepatic steatosis	73

**Figure 1:**
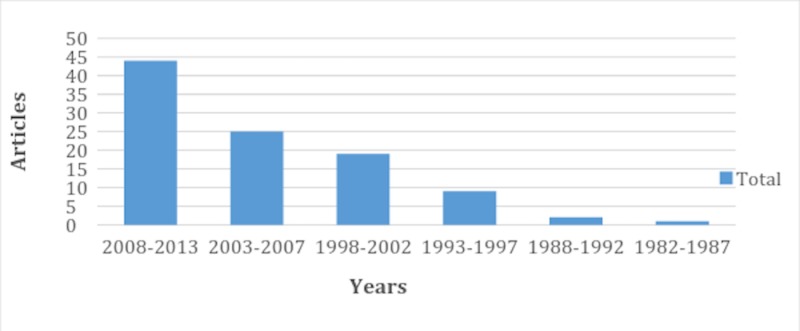
Five-year interval for the top 100 cited hepatic magnetic resonance imaging (MRI) articles

The total number of authors for 100 articles was 710 with a mean of 7.1 authors per article. The contribution of authors to the top 100 cited articles was reviewed and all authors contributing more than two articles to the highly cited articles are given in the supplementary table at the end (Table 3). All the highly cited articles were published during a span of 31 years from 1982 to 2013. The maximum number of articles were published during 2009 (14 articles), and for a five-year period, the maximum contribution was made by 2008-2013 (44 articles) (Figure 1).

The highest number of top 100 cited publications on hepatic MRI was from Radiology (30 articles) followed by European Radiology (14 articles), the American journal of Roentgenology, Radiographics, and Journal of Magnetic Resonance had seven articles each. Other journals like Investigative Radiology and Journal of Hepatology had four or more than four articles; 22 journals had only two or fewer articles. The impact factors for all the journals having articles in the top 100 cited articles ranged from 0.81 to 20.98 (Table 2).

**Table 2:** Total articles according to journal and their impact factor

Radiology	30 (4641)	6.867
European Radiology	14 (1936)	4.014
American Journal of Roentgenology	7 (734)	1.53
Radiographics	7 (798)	3.16
Journal of Magnetic Resonance Imaging	7 (729)	1.6
Investigative Radiology	6 (787)	4.887
Journal of Hepatology	4 (576)	11.05
Liver Transplantation	2 (261)	2.61
Hepatology	2 (452)	11.05
Journal of Clinical Gastroenterology	2 (294)	3.186
Academic Radiology	1 (177)	1.11
European Journal of Gastroenterology and Hepatology	1 (103)	2.152
Acta Paediatrica, International Journal of Paediatrics	1 (85)	0.92
European Journal of Radiology	1 (95)	1.78
Magnetic Resonance in Medicine	1 (84)	3.782
American Chemical Society Nano	1 (174)	13.334
Clinics in Liver Disease	1 (119)	2.09
Clinical Gastroenterology and Hepatology	1 (83)	7.896
Korean Journal of Radiology	1 (83)	1.803
Abdominal Imaging	1 (137)	0.65
Magnetic Resonance in Medical Sciences	1 (172)	0.77
Biomaterials	1 (182)	8.387
Medicine (United States)	1 (100)	2.133
Seminars in Liver Disease	1 (165)	5.1
European Journal of Nuclear Medicine and Molecular Imaging	1 (82)	5.383
British Journal of Radiology	1 (97)	1.16
World Journal of Gastroenterology	1 (137)	2.433
Journal of Clinical Oncology	1 (151)	20.982
Journal of Computer Assisted Tomography	1 (124)	0.81

**Figure 2:**
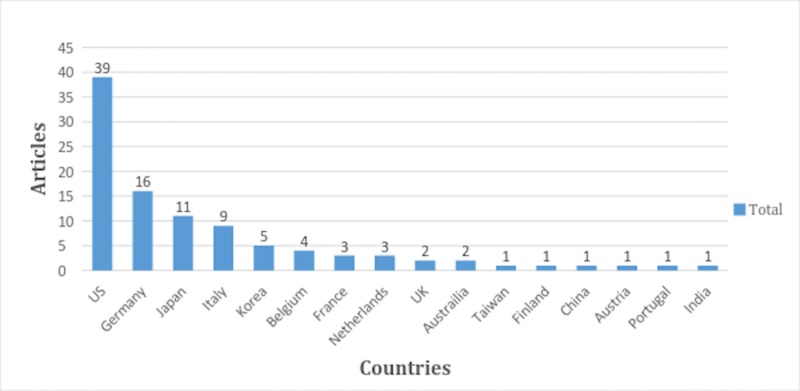
Articles according to the country of origin

A total of 16 countries contributed to the top 100 cited articles with the largest contribution made by the United States (39 articles). Other countries including Germany, Japan, Italy, and Korea published sixteen, eleven, nine and five articles respectively. Eleven countries had less than five articles in the top 100 cited articles on hepatic MRI (Figure 2).

**Figure 3:**
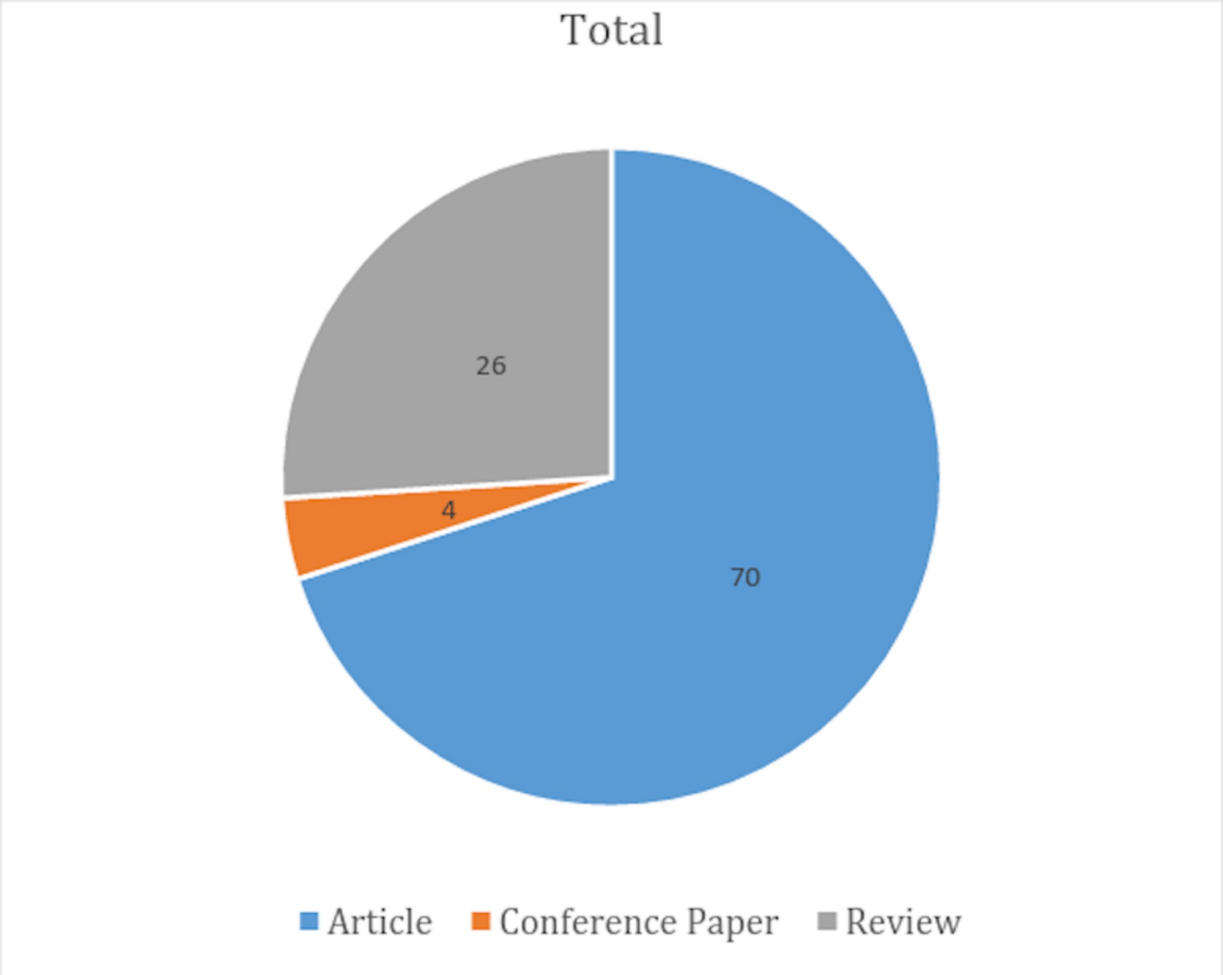
Type of manuscripts for the top 100 cited hepatic magnetic resonance imaging (MRI) articles

Of all the 100 articles, 70 articles were original articles, 26 were review articles while only four were conference papers (Figure 3).

In this extensive literature search, we found that the top two articles were about diffusion-weighted MRI primarily used for focal hepatic lesion detection. Both these articles were cited more than 300 times and were published in Radiology. A study of 160 patients showed that history, examination, and tumor markers are not sufficient for the diagnosis of the focal lesions, indicating the importance of hepatic MRI in the detection and differentiation of focal lesions [3]. Hepatic MRI also helps differentiate benign lesions like hepatic adenoma, hemangioma, and focal hyperplasia from malignant lesions making it an important diagnostic modality [4]. In our analysis, 70% of all the top cited articles were about the applications and characteristics of hepatic MRI for the diagnosis of hepatic nodules and metastasis. Further analysis showed that of the 15 articles with citations greater than 200, nine were about the application of hepatic MRI for the diagnosis of focal lesions and the comparison of MRI with other imaging techniques in diagnosing liver lesions. This high citation indicates that there is significant interest in the application of hepatic MRI for the diagnosis of focal lesions and metastasis, and it may be the most frequent use of a hepatic MRI.

Overall, it was found that a significantly large amount of work on hepatology imaging focused on the applications of different contrast agents and the comparison of different imaging techniques along with its use for the diagnosis of focal or metastatic lesions. In the list of top 100 cited articles, only eight articles discussed the applications of hepatic MRI for hepatic fibrosis, arterioportal shunts, and complications of cirrhosis. While only one article in the top 100 cited articles discussed the uses of hepatic MRI for the diagnosis of hepatic abscess and to delineate hepatic blood flow, highlighting the need for further research in these fields. This can also have important implications for the journal editors and stakeholders in identifying and evaluating scientific literature in the field of hepatology and radiology.

It is also necessary to acknowledge the possibility of publication bias toward these articles and that hepatology and radiology literature, like other medicine-related fields, may be tied to pharmaceutical industries [5]. Nonetheless, understanding the important features inherent to the highly cited articles would facilitate other researchers and investigators to publish effectively. We believe that this paper will also help to highlight the subspecialties, subtopics, and applications of hepatic MRI that have not been given enough consideration. For instance, from our analysis, there has not been significant research on the use of hepatic MRI for diagnosing some infectious etiologies, particularly abscess causing organisms, especially in the developing countries. We feel that this issue could have greater future representation in the list. Also with time, as the disease burden of liver diseases increases, we anticipate an increase in the use of hepatic MRI in general as a diagnostic modality [6]. Furthermore, more than 98% of all the articles were published in the developed countries and only one article was from India. This signifies the possibility that the application of MRI for the diseases like hepatic abscess and cirrhosis might have been under-reported indicating that research directions could be redefined.

Our analysis showed that the most highly cited articles were published in 29 different journals. More than half (n=51) of all the 100 top cited articles were published in the Radiology or European Radiology and American Journal of Roentgenology—all these journals were dedicated solely to the imaging techniques but had an impact factor less than seven. Our findings are interestingly in contrast to the application of Bradford’s law, a concept of bibliometric analysis suggested by Brookes [7-8]. Bradford’s law states that most investigators obtain their citations from few journals which are famous in their field of expertise and the impact and citation frequency gets weakened when the study deviates from their core journals. Consequently, this tendency leads to a higher number of citations stemming from a few journals with high impact factor [7-8]. However, our study indicates that there might be other factors responsible for the number of citations an article gets. One possibility would be a growing trend of publishing articles in journals specified for the specialty in contrast to journals with a high impact factor. The Journal of Clinical Oncology and the American Chemical Society contributed only one article to the list in spite of very high impact factors. Moreover, in concordance with many other bibliometric analyses, most of the articles (n=39) were from the United States [9-10]. Germany, Japan, Italy, Korea, and Belgium combined contributed 45 articles to the list of top 100 articles, indicating that most of the work on hepatic MRI has been done in developed countries. India which has a huge load of a population suffering from fatal hepatic diseases where approximately one in every five Indians has hepatic disease had only one article in the list of top 100 articles.

Further scrutiny of the top articles showed that most (n=44) articles of the top cited articles on hepatic MRI were published from 2008 to 2013. These findings can be explained by the fact that hepatology is a dynamic field in which there has been an immense advancement and a huge contribution to the literature recently. Furthermore, it signifies that clinicians and researchers prefer to rely more on the latest studies. But readers should consider that some elapsed time is required for the articles to gain a considerable number of citations and to achieve significant coverage. That is the possible reason for the relatively small number of articles in our list published during 2014 to 2017. While the absence of any article before 1982 in our list of top 100 cited articles suggests the limited usefulness of old articles in the modern era, or that not significant research was done around that time as MRI was invented only in 1977 [11-12]. However, this trend could be due to a combination of many factors like the quality of an individual study, database limitation to track older articles, lack of internet resources in the pre-1990s, and the fact that original work is often published in hard copies like textbooks rather than on online journals [13-14]. Figure 1 in our study interestingly depicts a downward trend in the number of articles over the years.

As with other bibliometric analyses, our study also has some potential limitations. First, we used only online search on one database, omitting articles which might have been published in textbooks forms and this might have also resulted in missing the articles published before 1980 as Scopus has been established lately. However, to make our search strategy more sensitive, we also did search on Google Scholar to cross-check the number of citations and to include the missing articles. Second, the inherent problems associated with citation analyses, such as the bias linked to rely on the total number of times an article is cited, must be noted as well. Furthermore, bibliometric analysis traditionally favors older articles and often omits latest articles usually from the last ten years. However, in our study, more than 50% of the top 100 cited articles on hepatic MRI were after 2000. Lastly, we used only Scopus library for our search; it is possible that our top 100 cited articles might have a different number of citations if we had used any other database like Google Scholar as a significant difference between various databases does exist.

## Conclusions

Overall, our study found that a large amount of work on hepatology imaging focused on the applications of different contrast agents and the comparison of different imaging techniques along with its use for the diagnosis of focal or metastatic lesions. Only one article in the top 100 cited articles discussed the uses of hepatic MRI for the diagnosis of hepatic abscess, and to delineate hepatic blood flow, highlighting the need for further research in these fields. Our study also indicates that most of the work on hepatic MRI has been done in developed countries and that most (n=44) articles of the top cited articles on hepatic MRI were published in the period between 2008 to 2013. Our analysis also showed that the most highly cited articles were published in 29 different journals with more than half (n=51) of all the 100 top cited articles published in the Radiology or European Radiology and American Journal of Roentgenology; all these journals were dedicated solely to the imaging techniques but had an impact factor less than seven. Our findings are in contrast to the application of Bradford’s law which states that most investigators obtain their citations from a few journals which are famous in their field of expertise and the impact and citation frequency gets weakened when the study deviates from their core journals. However, our study indicates that there might be other factors responsible for the number of citations an article gets. One possibility would be a growing trend of publishing articles in journals specified for the specialty in contrast to journals with a high impact factor.

## Appendices

**Table 3:** Table of the authors who contributed three or more articles to the list of top 100 cited articles

Authers	Total	First	Last	Others	Affiliation
Sirlin, CB	10	0	7	3	University of California San Diego, San Diego, USA
Grazioli, L	7	1	1	5	University of Brescia, Brescia, Italy
Lee, VS	6	1	2	3	New York University Medical Center, New York, USA
Reimer, P	6	4	0	2	Städtisches Klinikum Karlsruhe, Karlsruhe, Germany
Breuer, J	6	0	1	5	Schering, Müllerstraße Berlin, Germany
Balzer, T	5	0	1	4	Schering AG, Müllerstrasse Berlin, Germany
Krinsky, GA	6	2	0	4	New York University Medical Center, New York, USA
Taouli, B	4	3	1	0	Mount Sinai School of Medicine, New York, USA
Spinazzi, A	4	1	1	2	Bracco SpA, Milan, Italy
Weinreb, JC	4	0	2	2	New York University Medical Center, New York, USA
Reeder, SB	4	1	3	0	University of Wisconsin, Madison, USA
Reiser, MF	5	0	0	5	Ludwig-Maximilians-University Munich -MunichGermany
Hamilton, G	4	0	0	4	University of California San Diego, San Diego, USA
Kirchin, M	4	0	2	2	Bracco Imaging, Milan, Italy
Huppertz, A	4	1	0	3	Imaging Science Institute Charité, Berlin, Germany
Giovagnoni, A	4	0	1	3	Centro di Risonanza Magnetica, Ancona, Italy
Schoenberg, S	3	0	3	0	Ludwig-Maximilians-University Munich -MunichGermany
Schima, W	3	0	2	1	University of Vienna, Vienna, Austria
Siegelman, ES	3	2	1	0	University of Pennsylvania Medical Center, Philadelphia, USA
Pirovano, G	3	1	1	1	Bracco SpA, Milan, Italy
Schneider, G	3	1	1	1	University Hospital, Homburg/Saar, Germany
Stoker, J	3	0	2	1	University of Amsterdam, Amsterdam, Netherlands
Semelka, RC	3	2	1	0	University of North Carolina, Chapel Hills, USA
Lencioni, R	3	0	2	1	University of Pisa, Pisa, Italy
Murakami, T	3	0	0	3	Osaka University Graduate School of Medicine, Osaka, Japan
Morana, G	3	0	0	3	Ospedale Cà Foncello, Treviso, Verona, Italy
Stemmer, A	3	0	0	3	Siemens Medical Solutions, Magnetic Resonance, Erlangen, Germany
Rofsky, NM	3	0	0	3	New York University Medical Center, New York, USA
Tanimoto, A	3	2	0	1	School of Medicine, Keio University, Shinjuku-ku, Tokyo, Japan
Rummeny, E	5	0	2	3	Klinikum rechts der Isar, Technische Universität München, Munich, Germany
Taupitz, M	3	0	1	2	Humboldt-University of Berlin, Berlin, Germany
Zech, CJ	3	3	0	0	Munich University Hospitals-Grosshadern, Ludwig-Maximilians-University
Saini, S	3	0	1	2	Massachusetts General Hospital, Boston
Lee, JM	3	0	1	2	Seoul National University Hospital, Seoul, Korea
Bartolozzi, C	3	2	0	1	University of Pisa, Pisa, Italy
Aguirre, DA	3	0	0	3	University of California San Diego, San Diego, USA
Brown, JJ	3	1	1	1	The Johns Hopkins University School of Medicine, Baltimore, USA
Laniado, M	3	0	o	3	Abteilung für Radiologische Diagnostik, Tübingen, Germany
